# Risk factors of subclinical hypothyroidism and the potential contribution to miscarriage: A review

**DOI:** 10.1002/rmb2.12325

**Published:** 2020-03-18

**Authors:** Shuhei So, Fumiko Tawara

**Affiliations:** ^1^ Department of Reproductive and Perinatal Medicine Hamamatsu University School of Medicine Higashi‐ku Hamamatsu‐shi Shizuoka Japan; ^2^ Tawara IVF Clinic Suruga‐ku Shizuoka‐shi Shizuoka Japan

**Keywords:** fertility treatment, mild hypothyroidism, miscarriage, thyroid function, thyroid‐stimulating hormone

## Abstract

**Background:**

There is no clear cutoff value for thyroid‐stimulating hormone (TSH) level that defines subclinical hypothyroidism (SCH). Moreover, TSH levels can be affected by numerous factors. Although mild SCH has been implicated in miscarriage, the relationship between TSH levels and miscarriage remains unelucidated.

**Methods:**

We reviewed nine known risk factors affecting TSH levels and 28 studies investigating the potential association between mild SCH and miscarriage, examining whether these factors were considered.

**Main findings:**

Among 28 studies that examined whether mild SCH (TSH > 2.5 mIU/L) contributed to miscarriage, thyroid antibodies were measured in only 15. TSH measurement methods were described in 18 studies. Although the iodinated contrast medium used in hysterosalpingography (HSG) is stored in the body for a long time and is a risk factor for mild SCH, only one study described its potential impact on TSH levels. Nine studies, which concluded that mild SCH contributed to miscarriage, had thyroid status evaluated only after the onset of pregnancy, but not before.

**Conclusion:**

TSH levels can be significantly affected by patient demographics and health history, country of origin, and fertility treatment. It is important to consider these factors while evaluating mild SCH. It remains unclear how mild SCH contributes to miscarriage.

## 1 INTRODUCTION

Overt hypothyroidism has been reported to increase the risk of miscarriage, premature birth, hypertensive disorders of pregnancy, placental abruption, postpartum hemorrhage, and Cesarean section.^1^, ^2^, ^3^, ^4^, ^5^ Since overt thyroid dysfunction affects the mother and child, it must be adequately managed before and during pregnancy. Levothyroxine (LT4) treatment reduces the risk of miscarriage and preterm birth by normalizing thyroid function, including TSH levels.^6^

Subclinical hypothyroidism (SCH), a condition in which free thyroxine (FT4) is in the normal range but thyroid‐stimulating hormone (TSH) level is high, has also been reported to be a risk of miscarriage and premature birth.^7^ The American Thyroid Association (ATA) and the American Endocrine Society have published guidelines for managing thyroid function in women planning or becoming pregnant.^8^, ^9^ The ATA guideline describes that there is insufficient evidence that LT4 treatment contributes to an increased pregnancy rate in SCH women and thyroid autoantibody‐positive euthyroid women who are planning a natural pregnancy; however, SCH women are recommended to consider a small amount of LT4 to reduce the risk of hypothyroidism after pregnancy. In contrast, when infertility treatment is performed in IVF or ICSI, controlling TSH levels to <2.5 mIU/L by LT4 is recommended to improve ART outcomes. During pregnancy, the use of a population‐based reference for the upper limit of TSH is suggested. If a population‐based reference cannot be obtained, it is recommended that the reference value for pregnant women be 0.5 mIU/L lower than the upper limit of nonpregnant women for each TSH measurement method. In particular, pregnant women with thyroid autoantibodies are recommended to consider LT4 supplementation with a target TSH level < 2.5 mIU/L.

Many studies have assessed the effect of mild SCH in early pregnancy on adverse events of pregnancy with a TSH cutoff value > 2.5 mIU/L.^10^, ^11^, ^12^, ^13^, ^14^, ^15^, ^16^, ^17^, ^18^, ^19^, ^20^, ^21^, ^22^, ^23^, ^24^, ^25^, ^26^, ^27^, ^28^, ^29^, ^30^, ^31^, ^32^, ^33^, ^34^, ^35^, ^36^, ^37^ The secretion of TSH is not only strongly influenced by various health factors, but is also known to have different baseline values due to differences in age and race.^38^ There is still a question as to whether TSH > 2.5 mIU/L is effective as a common global index for mild SCH and whether LT4 treatment is needed in all cases where TSH exceeds this value.

First, we review the risk factors that affect TSH secretion. Next, we review previous studies that examined whether mild SCH both prior to and during pregnancy contributes to miscarriage, and then discuss the relationship between the timing of evaluation for mild SCH and the miscarriage rate. Finally, we try to conclude when and how to evaluate patients who are or may become pregnant for mild SCH.

## 2 RISK FACTORS AFFECTING TSH

TSH is a sensitive marker of thyroid function. Factors affecting TSH levels should be considered when assessing the presence and potential impact of mild SCH. Here, we review the following factors: thyroid antibodies, age, body mass index (BMI), iodine intake, daily and seasonal changes, TSH measurement methods, hysterosalpingography, ovarian stimulation, and human chorionic gonadotropin (hCG) production (Table 1).

**TABLE 1:** Risk factors affecting TSH

Risk factors	TSH response	Candidate mechanisms
Advanced age	Increase	Age‐dependent changes of the HPT axis
Obesity	Increase	Stimulation of TSH secretion by leptin
Iodine intake	Increase	Wolff‐Chaikoff effect
Low temperature (Winter)	Increase	HPT axis regulates thermogenesis
Hysterosalpingography	Increase	Iodinated contrast medium (Wolff‐Chaikoff effect)
Ovarian stimulation	Increase	High estrogen levels → Rise in TBG → Transient decrease in FT4 → Rise in TSH
High temperature (Summer)	Decrease	HPT axis regulates thermogenesis
Food intake	Decrease	Suppression of TSH secretion by somatostatin
hCG production at early pregnancy	Decrease	Cross‐reactivity between TSH and hCG
TSH measurement methods	Increase/decrease	Different measurement principle
Ethnicity	Increase/decrease	Differences in genetic background or food culture
Daily change	Increase/decrease	Circadian rhythm

Abbreviations: FT4, free T4; hCG, human chorionic gonadotropin; HPT, Hypothalamic‐pituitary‐thyroid; TBG, thyroxine‐binding globulin; TSH, thyroid‐stimulating hormone.

### 2.1 Thyroid antibodies

Anti‐thyroid peroxidase antibody (TPOAb) and anti‐thyroglobulin antibody (TgAb), which are thyroid autoantibodies, are risk factors for SCH and have been reported to be prevalent in infertile women and miscarriage patients.^38^, ^39^ A meta‐analysis by Thangaratinam et al^40^ indicated that thyroid autoantibodies contribute to increased miscarriage rates. The mechanism by which thyroid autoantibodies contribute to miscarriage is unclear, but Negro et al^41^ pointed out that the high miscarriage rate among women with TPOAb may be due to the onset of SCH during pregnancy.

Several genes related to thyroid autoimmunity have been identified: HLA‐DRB1 locus,^42^ cytotoxic T‐lymphocyte‐associated protein 4,^43^, ^44^ CD40,^45^ protein tyrosine phosphatase‐22,^46^ thyroglobulin,^47^, ^48^ TSH‐receptor,^49^ and Vitamin D receptor gene.^50^ The disease penetrance of mutations in these genes varies widely with ethnicity.^42^, ^51^ Consistent with this, there is a racial difference in the prevalence of thyroid antibodies.^38^ According to a nationwide survey in the United States, the prevalence of TPOAb in Caucasian women is 12.6% (ages 20‐29), 14.3% (ages 30‐39), and 19.5% (ages 40‐49). The prevalence in African‐American women (in the same age brackets, respectively) is 4.8%, 6.3%, and 8.3%, and in Hispanic women 14.0%, 16.8%, and 18.9%. The prevalence of TgAb in Caucasian women is 10.1%, 14.9%, and 18.0%; in African‐American women 3.0%, 4.1%, and 5.0%; and in Hispanic women 11.6%, 13.0%, and 14.9%. The prevalence of thyroid autoantibodies in African‐Americans is lower than in Caucasians; the prevalence of TPOAb and TgAb is similar in each ethnicity. Conversely, the prevalence of TgAb in Japan is significantly higher than that of TPOAb (16.5% vs 9.4% in Takeda et al,^52^ and 29.4% vs 15.0% in Noso et al^53^). Similar results have been obtained in our previous studies of women undergoing infertility treatment (11.5% TgAb vs 9.3% TPOAb with TSH < 2.5 mIU/L, 17.0% vs 11.7% with TSH 2.5‐3.5 mIU/L^33^). Although TPOAb is thought to be superior for identifying thyroid autoimmunity than TgAb,^54^, ^55^ both are risk factors for SCH. For ethnicities with a high TgAb frequency, such as Japanese, TgAb measurement should probably be the first choice.

### 2.2 Age and BMI

TSH is known to be positively correlated with both age^38^, ^52^, ^56^, ^57^ and body mass index (BMI).^58^, ^59^ Therefore, mild SCH may be more frequent in older and overweight women. In fact, Surks and Hollowell^56^ showed that among thyroid autoantibody‐negative women of childbearing age, TSH values of 2.5‐4.5 mIU/L are found in 6.5% of women aged 20‐29 years, in 9.5% aged 30‐39 years, and in 11.5% aged 40‐49 years. Chakraborti et al^60^ reported that aging alters pituitary TSH secretion. They showed that a thyrotropin‐releasing hormone (TRH)‐stimulated TSH peak was reduced in elderly compared with younger subjects. Using the Korean national database, Park et al^61^ reported that the relationship between age and TSH is a U‐shaped function, with minimum TSH values in the range of 30‐50 years old. A U‐shaped association between age and urinary iodine concentrations was also observed. This result suggests that changes in age‐dependent iodine intake may affect age‐dependent TSH levels.

Knudsen et al^59^ reported that the prevalence of a BMI > 30 is higher in subjects with serum TSH 2.0‐3.6 mIU/L (odds ratio (OR) 1.20 [0.94‐1.55]) and TSH > 3.6 mIU/L (OR 2.13 [1.44‐3.14]) compared to the subjects with serum TSH 1.0‐1.99 mIU/L. Furthermore, weight loss contributes to TSH level reduction.^62^, ^63^ One possible mechanism by which obesity causes an increase in TSH level may be that adipose tissue promotes secretion or synthesis of TSH.^64^ Actually, leptin secreted from adipose cells is known to promote TRH secretion.^65^, ^66^

### 2.3 Iodine intake

Foods with high iodine content, such as seaweed, have a significant effect on TSH levels.^67^ When adults with negative thyroid autoantibodies ingested 35 mg/d of iodine, TSH values increased significantly from 2.15 ± 0.32 to 4.31 ± 0.57 mIU/L, indicating the Wolff‐Chaikoff effect,^68^ and returned to the basal value after the withdrawal of iodine (TSH 2.33 ± 0.47 mIU/L).^67^ Thus, intake of food with a high iodine content before blood collection is directly reflected in the TSH value.^69^ Iodine intake can be assessed by urine iodine concentration (UIC). Candido et al^70^ measured UIC in women during early pregnancy and reported that individuals from Japan, Canada, and Venezuela may be iodine‐sufficient, while those in the UK, Turkey, Austria, Spain, Iran, Bangladesh, and Australia may be iodine‐insufficient. However, although Japan is an iodine‐sufficient area, UIC < 150 μg/mL, which is defined as mildly iodine deficient,^8^ is observed in approximately 30% of pregnant women in Japan.^71^ Zimmermann and Delange^72^ reviewed pregnant women's iodine intake in several European countries and found that individuals in Sweden and Switzerland were iodine‐sufficient, while those in Belgium, Denmark, France, Germany, Hungary, Ireland, Italy, and Turkey were iodine‐insufficient.

Even within the same country, iodine intake may vary depending on food culture differences. In China, Li et al^73^ found regional differences in median urinary iodine concentrations of 83.5 μg/L, 242.9 μg/L, and 650.9 μg/L in people living in Panshan, Zhangwu, and Huanghua, respectively, and the frequency of TSH > 4.8 was significantly positively correlated with UIC (1.32%, 8.49%, and 15.38%, respectively). Völzke et al^74^ suggested that the TSH reference values in the iodine‐sufficient group and ‐insufficient group may be different. In addition, the results of the nationwide survey in the United States indicated that the basic value of TSH may differ depending on race.^38^ This study found that African‐American women had significantly lower TSH levels than Caucasian women (1.15 ± 0.02 vs 1.55 ± 0.03) and lower frequency of SCH (TSH > 4.5 mIU/L) (1.5% vs 6.6%). Caucasians have been reported to have higher iodine intake than African‐Americans, so the difference in TSH levels among races may be due to the differences in food culture.^75^

### 2.4 Daily and seasonal changes

TSH conforms to circadian rhythm, and TSH secretion increases up to 1.5 times at nighttime compared with daytime.^76^ This circadian rhythm is age‐related,^77^ shows large interindividual variability,^78^ and has been reported to be disturbed by an irregular sleep‐wake cycle.^79^ In addition, eating is known to act on the pituitary‐thyroid axis and reduce TSH secretion.^80^ Nair et al^81^ reported that TSH secretion was significantly reduced 2 hours postprandially compared with the fasting state in the same subject: 2.42 ± 1.49 to 1.79 ± 1.10 for euthyroid subjects (n = 19); 7.53 ± 1.50 to 5.35 ± 1.40 for SCH subjects (n = 20); and 66.93 ± 17.83 to 61.22 ± 16.41 for overt hypothyroid subjects (n = 18). Although leptin secretion induced by food intake contributes to increased TSH (see the previous section on age and BMI), somatostatin, which also rises after food intake, acts as a negative regulator for leptin and inhibits TSH secretion.^82^, ^83^ As a result, TSH levels tend to decrease just after food intake. Through such hormonal action, fasting^84^ and night eating^85^ affect the circadian rhythm of TSH. Thus, TSH values and the frequency of detection of mild SCH may vary depending on the timing of blood collection.

The hypothalamic‐pituitary‐thyroid axis is known to regulate thermogenesis.^86^ Seasonal variability of TSH levels has been reported in countries with four seasons such as Japan and Korea.^87^, ^88^ Yoshihara et al^87^ reported clinical data from more than 1.6 million individuals, indicating that TSH levels tend to be lower in summer than in winter: the median TSH value was 1.38 in spring (March‐May); 1.31 in summer (Jun‐Aug); 1.41 in autumn (Sep‐Nov), and 1.46 in winter (Dec‐Feb). Consistent with this, Kim et al^88^ reported a 1.4‐fold increase in SCH frequency in winter‐spring compared with that of summer‐fall. Thus, in areas with a wide annual temperature range, it may be necessary to consider seasonal temperature variations, especially when evaluating for mild SCH. These results suggest that when TSH > 2.5 mIU/L is set as the universal cutoff value, there may be a difference in the prevalence of mild SCH in warm vs cold regions.

### 2.5 TSH measurement methods

Currently, third‐generation TSH assays are the main method for screening for primary hypothyroidism. Most of these assays are two‐site “sandwich” immunoassays that use an enzyme or chemiluminescent label.^89^, ^90^ Unfortunately, the differences across these measurement methods affect the measured value of TSH.^91^ Therefore, multicenter research studies employing different TSH measurement methods may produce results that are incomparable. In addition, it is difficult to compare data from published studies that used different third‐generation measurement methods. Because of these issues, the IFCC C‐STFT (International Federation of Clinical Chemistry and Laboratory Medicine, Committee of Standardization of Thyroid Function Tests) has begun to develop reference measurement systems to establish traceability and comparability of TSH assays.^92^

### 2.6 Hysterosalpingography

In hysterosalpingography (HSG), an oil‐based or water‐soluble iodine‐containing contrast medium is used. It has been reported that patients receiving oil‐based contrast media have a higher cumulative pregnancy rate after HSG than patients receiving water‐based contrast media.^93^, ^94^, ^95^ However, oil‐based contrast media have a longer retention period, which significantly increases the frequency of SCH compared with water‐soluble contrast media.^94^, ^96^, ^97^ Kaneshige et al^96^ found that oil‐based contrast media had a retention period of more than 6 months. When oil‐based contrast media were introduced to patients with TSH < 2.5 mIU/L, the patients' TSH values exceeded 2.5 mIU/L (in 72.7% after 1 month, in 52.4% after 2 months, and in 46.7% after 6 months). Our group reported that the frequency of SCH (TSH > 2.89 mIU/L) was 22.6% after 1 month and 24.4% after 2‐6 months from HSG with an oil‐based contrast agent.^94^ We also found that the frequency of SCH is significantly lower in HSG patients receiving water‐soluble than oil‐based contrast media: 9.5% after 1 month, 3.6% after 2‐6 months. This means that the type of contrast medium used and the timing of HSG affect the frequency of SCH. Furthermore, the use of oil‐soluble iodinated contrast medium may cause fetal hypothyroidism.^98^ However, there are currently no data to suggest that the miscarriage rate increases after HSG with an oil‐based contrast medium.

### 2.7 Ovarian stimulation

Elevated serum estradiol (E2) concentration due to ovarian stimulation with assisted reproductive technology (ART) is known to affect the hypothalamic‐pituitary‐thyroid axis and might be a risk factor for SCH (high estrogen levels → rise in thyroxine‐binding globulin (TBG) → transient decrease in FT4 → rise in TSH^99^). Muller et al^100^ reported that the TSH value before ovarian stimulation was 2.3 ± 0.3 mIU/L and increased significantly to 3.0 ± 0.4 mIU/L thereafter. Similarly, Poppe et al^101^ reported a significant increase in TSH values after ovarian stimulation (1.8 ± 0.9 mIU/L pre‐stimulation vs 3.3 ± 2.4 mIU/L post‐stimulation). They also found that the increase in TSH values after ovarian stimulation was higher in TPOAb‐positive than in TPOAb‐negative individuals. Benaglia et al^102^ reported that 35% of patients with initial TSH values < 2.5 mIU/L had TSH levels higher than 2.5 mIU/L after ovarian stimulation. Therefore, the question of whether the presence of mild SCH before or after ovarian stimulation contributes to adverse pregnancy outcomes, including miscarriage, is of special importance in patients undergoing ART.

### 2.8 Changes in thyroid function owing to pregnancy

Gestational age and hormones that regulate pregnancy, including estrogen and hCG, affect thyroid function, including TSH levels, during pregnancy.

Estrogen, which increases during pregnancy, contributes to an increase in thyroxine‐binding globulin (TBG) levels.^99^ TBG begins to increase from early pregnancy and is high until the end of pregnancy. Serum TBG levels in pregnant women are approximately 2.5 times higher than those in nonpregnant women. The increased TBG level promotes thyroid hormone synthesis.

Human chorionic gonadotropin (hCG) has a high structural homology to TSH and is known to exhibit TSH‐like activity.^99^, ^103^, ^104^ Serum TSH shows a transient decline at 9‐10 weeks of gestation when serum hCG is at its maximum (~100 000 IU/L) and a gradual increase after the second trimester. Glinoer^99^ estimated that as hCG rises to 1000 IU/L, TSH shows a 0.1 mIU/L decrease. A decrease in TSH level is even more pronounced in twin pregnancies where hCG is higher than in single pregnancies.^105^ The trimester‐specific range is used as the TSH reference value during pregnancy.^8^, ^9^, ^99^ The ATA guideline recommends using a population‐based reference range, defined based on local population data, as the upper limit of TSH during pregnancy.^8^ If the population‐based reference cannot be obtained, it is recommended that the reference value for pregnant women be 0.5 mIU/L lower than the upper limit of nonpregnant women for each TSH measurement method.

## 3 DOES MILD SCH CONTRIBUTE TO MISCARRIAGE?

We first selected 28 studies from the PubMed search of the relevant literature using TSH > 2.5 mIU/L as the cutoff value and the miscarriage rate as the outcome (Tables 2 and 3: one study^31^ is included in both Tables 2 and 3). Then, we examined whether the factors affecting TSH values mentioned above were considered in the studies. We evaluated whether the reader could interpret the results considering these confounding factors, not whether the authors of the studies accounted for them. Thyroid autoantibodies were measured in 15 of the 28 studies (53.6%); seven of these 15 studies measured TPOAb only (46.7%), and eight measured both TPOAb and TgAb (53.3%). The reason only TPOAb was measured in some studies is probably that the ATA recommends the measurement of TPOAb as the first choice for detection of thyroid autoimmunity.^8^

**TABLE 2:** Characteristics of studies involving mild SCH patients with TSH > 2.5 mIU/L cutoff value

First author (Year) Country Study type	Patient background (Fertility treatment)	Euthyroid group (age) SCH group (age)	SCH judgement	Risk factors	Miscarriage rate
Baker (2006) ^10^ USA Retrospective cohort	Infertility women (Fresh‐ET/Frozen‐ET)	≤2.5: n = 272 (36.0 ± 4.2) >2.5: n = 92 (35.4 ± 4.7)	w/in 1 y before IVF	Method: CLIA TAI: Not measured HSG: Not shown	No difference
Negro (2010) ^11^ Italy Prospective cohort	Pregnant women (None)	<2.5: n = 3481 (28.7 ± 5) 2.5‐5.0: n = 642 (29.2 ± 5)	w/in 11 wk of gestation	Method: Not shown TAI: TPOAb HSG: Not shown	SCH patients ↑
Reh (2010) ^12^ USA Retrospective cohort	Infertility women (Fresh‐ET)	<2.5: n = 807 (36.7 ± 4.8) ≥2.5: n = 248 (37.1 ± 4.7)	w/in 1 y before IVF	Method: Not shown TAI: Not measured HSG: Not shown	No difference
Michalakis (2011) ^13^ USA Retrospective cohort	Infertility women (Fresh‐ET)	0.4‐2.5: n = 842 (34.9 ± 4.3) >2.5‐4.0: n = 278 (35.1 ± 4.5)	w/in 6 wk before IVF	Method: Not shown TAI: Not measured HSG: Not shown	No difference
Wang (2012) ^14^ China Prospective cohort	Pregnant women (Unknown)	<2.5: n = 542 (27.8 ± 0.1)* ≥2.5 n = 168 (28.1 ± 0.3)*	w/in 12 wk of gestation	Method: CLIA TAI: Not measured HSG: Not shown	SCH patients ↑
Busenelli (2013) ^15^ Italy Case‐control	Infertile women (Fresh‐ET)	≤2.5: n = 274 (34.9 ± 3.5) >2.5: n = 137 (35.0 ± 3.3)^a^	Before ovarian stimulation	Method: CLIA TAI: TPOAb/TgAb^b^ HSG: Not shown	No difference
Chai (2014) ^16^ China Retrospective cohort	Infertility women (Fresh‐ET)	<2.5: n = 508 ≥2.5: n = 119 In total subjects: 35.3 ± 3.2	Before ovarian stimulation	Method: CLIA TAI: TPOAb/TgAb HSG: Not shown	No difference
Mintziori (2014) ^17^ Greece Retrospective cohort	Infertility women (Fresh‐ET)	0.5‐2.5: n = 120 (37[7]^†^) >2.5‐4.5: n = 38 (37[8]^†^)	Before ovarian stimulation	Method: CLEIA TAI: TPOAb/TgAb HSG: Not shown	No difference
Liu (2014) ^18^ Liaoning Province, China Prospective cohort	Pregnant women^c^ (None)	0.29‐<2.5: n = 1061 (29.9 ± 3.5) 2.5‐10.0: n = 755 (29.5 ± 3.4)	w/in 8 wk of gestation	Method: ECLIA TAI: TPOAb/TgAb HSG: Not shown	SCH patients ↑
Taylor (2014) ^19^ UK Retrospective cohort	Pregnant women (Unknown)	0.2‐2.5: n = 199 2.51‐4.5: n = 151 In total subjects: age range 18‐45	First trimester	Method: Not shown TAI: Not measured HSG: Not shown	No difference
Green (2015) ^20^ USA Retrospective cohort	Infertility women (Fresh‐ET/Frozen‐ET)	≤2.5: n = 1028^d^ >2.5: n = 571^e^ (34.9 ± 4.3)	8 d after ET	Method: ECLIA TAI: Not measured HSG: Not shown	No difference^f^
Hammond (2015) ^21^ USA Retrospective cohort	Infertility women (non‐ART^g^/Fresh‐ET/ Frozen‐ET)	<2.5: n = 71 ≥2.5: n = 23^e^ In the total subjects: 33.0 ± 4.1	w/in 6 wk of gestation	Method: CLIA TAI: TPOAb HSG: Not shown	No difference
Karmon (2015) ^22^ USA Retrospective cohort	Infertility women (IUI)	0.4‐2.5: n = 1079 (34.8 ± 4.4) 2.5‐4.99: n = 398 (35.1 ± 4.5)	Before IUI	Method: CLIA/ECLIA TAI: Not measured HSG: Not shown	SCH patients ↓^h^
Coelho Neto (2016) ^23^ Brazil Retrospective cohort	Infertility women (Fresh‐ET)	<2.5: n = 455 (35[7]^†^) 2.5‐4.0: n = 162 (35[6]^†^)	Unknown	Method: 3rd generation TAI: Not measured HSG: Not shown	No difference
Gingold (2016) ^24^ USA Retrospective cohort	Infertility women (Fresh‐ET)	0.5‐2.5: n = 773 (37.0 ± 4.6) 2.5‐5.0: n = 317 (37.4 ± 4.4)	w/in 2 wk before IVF	Method: Not shown TAI: Not measured HSG: Not shown	No difference
Unuane (2016) ^25^ Belgium Retrospective cohort	Infertility women (IUI)	<2.5: n = 2696 ≥2.5: n = 447 In total subjects: age range 19‐46	Before IUI	Method: ECLIA TAI: TPOAb HSG: Not shown	No difference
Seungdamrong (2017) ^26^ USA Secondary analysis of RCT	Infertility women (non‐ART^g^)	0.2‐<2.5: n = 1146 (30.5 ± 4.5) 2.5‐5.5: n = 322 (30.6 ± 4.5)	Before fertility treatment	Method: CLEIA TAI: TPOAb HSG: Not shown	No difference
Uchida (2017) ^27^ Japan Retrospective cohort	RPL women (Unknown)	0.3‐2.5: n = 261 (35.9 ± 4.3) >2.5‐4.5: n = 56 (35.8 ± 3.9)	Before pregnancy	Method: ECLIA TAI: Not measured HSG: Not shown	No difference
Hernandez (2018) ^28^ Spain Retrospective study	Pregnant women (Unknown)	<2.5: n = 1448 2.5‐5.0: n = 470 In the total subjects: 30.1 ± 5.7	9‐12 wk of gestation	Method: ECLIA TAI: TPOAb HSG: Not shown	SCH patients ↑
Tuncay (2018) ^29^ Turkey Retrospective cohort	Infertility women (IUI)	0.40‐2.49: n = 233 (29.3 ± 4.4) 2.5‐4.99: n = 69 (29.1 ± 4.4)	Before ovarian stimulation	Method: ECLIA TAI: Not measured HSG: Not shown	No difference
Jin (2019) ^30^ China Retrospective cohort	Infertility women (Fresh‐ET)	≤2.5: n = 830 (31.5 ± 4.6) >2.5: n = 355 (31.0 ± 4.9)	Before ovarian stimulation	Method: ECLIA TAI: TPOAb HSG: Not shown	No difference
Kianpour (2019) ^31^ Iran Prospective cohort	Pregnant women^c^ (Unknown)	0.1‐2.5: n = 323 >2.5: n = 89^i^ In the total subjects: 28.8 ± 4.9	5‐14 wk of gestation	Method: Not shown TAI: TPOAb HSG: Not shown	SCH patients ↑
Li (2019) ^32^ China Case‐control	Pregnant women (Unknown)	0.4‐2.5: n = 1684 2.5‐4.87: n = 421 In total subjects: age range 19‐40	4‐9 wk of gestation	Method: ECLIA TAI: TPOAb/TgAb HSG: Not shown	SCH patients ↑
So (2019) ^33^ Japan Retrospective cohort	Infertility women (non‐ART^g^/Fresh‐ET/ Frozen‐ET)	<2.5: n = 1249 (34.3 ± 4.6) 2.5‐3.5: n = 230 (34.8 ± 4.6)	First visit at the clinic/before pregnancy	Method: ECLIA TAI: TPOAb/TgAb HSG: Before HSG^j^	No difference
Turgay (2019) ^34^ Turkey Retrospective cohort	Infertility women (IUI)	0.5‐2.49: n = 118 (28[23‐44]^‡^) 2.5‐5.0: n = 38 (30[22‐36]^‡^)	w/in 3 mo before IUI	Method: CLEIA TAI: TgAb/TPOAb HSG: Not shown	No difference

Note

The data are presented as the mean ± standard deviation, median [interquartile range] ^†^, median [min‐max] ^‡^, or mean ± standard error *.

Abbreviations: ART, assisted reproductive technology; CLEIA, Chemiluminescent enzyme immunoassay; CLIA, Chemiluminescence immunoassay; ECLIA, Electro‐chemiluminescence immunoassay; ET, embryo transfer; hCG, human chorionic gonadotropin; HSG, hysterosalpingography; intrauterine insemination; IUI, intrauterine insemination; IVF, in vitro fertilization; LT4, levothyroxine; RPL, recurrent pregnancy loss; SCH, subclinical hypothyroidism; TAI, thyroid autoimmunity.

^a^ Patients with TSH > 2.5 mIU/L received LT4 before ovarian stimulation, but TSH level measurement did not follow.

^b^ Thyroid antibody test was performed in the study group.

^c^ Urinary iodine concentration was measured.

^d^ <0.5 (35.8 ± 4.5), 0.5‐<1.0 (35.7 ± 3.7), 1.0‐<1.5 (35.1 ± 4.3), 1.5‐<2.0 (35.3 ± 4.2), 2.0‐2.5 (35.3 ± 4.0).

^e^ All subjects had TSH < 2.5 mIU/L before conception.

^f^ The miscarriage rates were compared among each TSH group.

^g^ Non‐ART includes fertility medication and IUI.

^h^ Miscarriage rate of patients with TSH 2.5‐4.99 is lower compared to that of patients with TSH 0.4‐1.36.

^i^ Sixty‐one out of 89 pregnant women who had TSH > 2.5 were treated with LT4.

^j^ Thyroid function tests were performed before HSG.

**TABLE 3:** Characteristics of studies involving mild SCH patients receiving LT4 treatment

First author (Year) Country Study type	Patient background (Fertility treatment)	Control group	Study group	I. Start of LT4 II. Target TSH level III. Follow‐up of TSH	Risk factors	Miscarriage
Bernardi (2013) ^35^ USA Retrospective cohort	PRL women (Unknown)	>2.5 w/o LT4 n = 39 (32.7 ± 4.3)	>2.5 with LT4 n = 221 (32.6 ± 3.9)	I. Before pregnancy II. <2.5 mIU/L, III. Yes	Method: Unknown TAI: Not shown HSG: Not shown	No difference^a^
Ma (2016) ^36^ China RCT^b^	Pregnant women (Unknown)	>2.5 w/o LT4 n = 996 (29[4] ^†^)	>2.5 with LT4 n = 675 (31[4] ^†^)	I. Early pregnancy II. Unknown, III. Yes	Method: CLIA TAI: TPOAb/TgAb HSG: Not shown	Study group ↓
Maraka (2017) ^37^ USA Retrospective cohort	Pregnant women (Unknown)	2.5‐10.0 w/o LT4 n = 4562 (31.3 ± 5.2)	2.5‐10.0 with LT4 n = 843 (31.7 ± 4.7)	I. First‐Second trimester II. Unknown, III. Yes	Method: Not shown TAI: Not shown HSG: Not shown	Study group ↓
Kianpour (2019) ^31^ Iran Prospective cohort^c^	Pregnant women^d^ (Unknown)	>2.5 w/o LT4 n = 28	>2.5 with LT4 n = 61	I. 5‐14 weeks of gestation II. Unknown, III. No	Method: Not shown TAI: TPOAb HSG: Not shown	Study group ↓

Note

The data are presented as the mean ± standard deviation, as the median [interquartile range] ^†^.

Abbreviations: CLIA, Chemiluminescence immunoassay; ECLIA, Electro‐chemiluminescence immunoassay; ET, embryo transfer; HSG, hysterosalpingography; intrauterine insemination; IVF, in vitro fertilization; LT4, levothyroxine; RCT, randomized controlled trial; RPL, recurrent pregnancy loss; SCH, subclinical hypothyroidism; TAI, thyroid autoimmunity.

^a^ Euploid miscarriages were statistically significantly less frequent in the SCH group. Trisomic miscarriages were more frequent in the SCH group.

^b^ Multicenter single blind RCT.

^c^ A comparison of miscarriage rates between euthyroid and SCH is shown in Table 2.

^d^ Urinary iodine concentration was measured.

Since the women studied were primarily of childbearing age, mostly in their thirties, we did not expect age to be a significant factor in the development of mild SCH in these particular studies. However, the contribution of mild SCH to miscarriages may increase with advancing age and obesity as they are known to be risk factors.^106^ Therefore, these risk factors need to be considered in this context.

Iodine intake and average temperature can be assessed according to the country in which the study was conducted. The iodine intake status of a country or region can be found in the WHO report.^107^ Whether to use different TSH cutoff points for countries with sufficient vs insufficient iodine intake, or for regions with high vs low average temperature, is an interesting question that requires further consideration. Only two studies^18^, ^31^ out of the 28 studies included measurement of urinary iodine. In common medical practice, however, it is not practical to assess urinary iodine in all cases.

Our review has established that the measurement method for TSH affects its value. This means that when TSH > 2.5 mIU/L is used as the cutoff value, the frequency of mild SCH diagnosis might vary due to the differences in TSH measurement methods. Therefore, the notation of the TSH measurement method is important and should be included in research studies. Of the 28 studies reviewed here, 19 studies reported a description of the TSH measurement method (67.9%). One study only described the measurement method as “third‐ generation.” Currently, most TSH measurement methods are of the third generation. Since there are several types of third‐generation measurement, merely describing the method as “third‐generation” is insufficient. Furthermore, it should be noted that even if the TSH measurement principle is the same but the assay kits' components are different, TSH values may be affected.

HSG using iodine contrast media is one of the causes of transient mild SCH. However, only one study (our study) considered the timing of TSH measurement relative to HSG. In addition, there was no report other than ours that indicated the type of contrast medium (oil‐soluble or water‐soluble) used. As mentioned earlier, when an oil‐based contrast medium is used, the storage period in the body is more than half a year.^96^ Therefore, if a patient begins intrauterine insemination (IUI) or in vitro fertilization (IVF) treatment several months after an HSG, the retention of iodine may cause transient mild SCH. Thus, at least the type of contrast medium used and the time relationship between TSH measurement and HSG should be indicated in research studies.

An increase in serum E2 concentration due to ovarian stimulation in IVF contributes to an increase in TSH concentration, whereas an increase in serum hCG concentration in early pregnancy leads to a decrease in TSH concentration. Therefore, information on the time when TSH was measured is important in evaluating mild SCH. Of the 28 studies, 27 described clearly the timing of TSH measurement relative to fertility treatment or weeks of pregnancy (96.7%). Among 25 studies comparing miscarriage rates in euthyroid women (TSH < 2.5 mIU/L) and women with mild SCH (TSH > 2.5 mIU/L), six have found an increase in miscarriage rates in the latter group (Table 2). Notably, all of them were diagnosed as having mild SCH based on TSH values after the onset of pregnancy. Conversely, in women who underwent IUI, Karmon et al^22^ reported a lower miscarriage rate in women with mild SCH than in euthyroid women. However, this study evaluated mild SCH before ovarian stimulation, and TSH values in early pregnancy are unknown. Taken together, these results suggest that the presence of mild SCH in early pregnancy may be a stronger contributor to miscarriage than the presence of mild SCH when planning pregnancy. In addition, of the four studies that evaluated the effect of LT4 on mild SCH, three studies showed a reduction in miscarriage rate with LT4 treatment and they included patients with mild SCH in early pregnancy. The fourth study that did not show any benefit from LT4 treatment included patients with mild SCH before pregnancy. Since there is no compelling evidence that developing mild SCH before pregnancy contributes to miscarriage, there is also no evidence to support the recommendation that all patients with TSH > 2.5 mIU/L should receive LT4 treatment before pregnancy. TSH in women during early pregnancy is lower than that in non‐pregnancy women who are planning pregnancy. Therefore, if the same cutoff value (TSH 2.5 mIU/L) is used in nonpregnant and pregnant women, aggressive treatment of nonpregnant women with TSH > 2.5 mIU/L may be an overtreatment among women with low‐risk miscarriage. Thus, changes in TSH levels before and after pregnancy make it difficult to evaluate SCH in women who wish to become pregnant, and the timing of starting LT4 supplementation is being discussed.

Whether mild SCH contributes to adverse pregnancy events, including miscarriage, is a topic of great interest, and many studies conducted globally have addressed it. In order to allow other researchers to accurately compare and evaluate their data across studies, it is necessary to provide sufficient information on the exact method and timing of the evaluation for mild SCH.

## 4 CONCLUSION

TSH secretion is influenced by many factors. When evaluating mild SCH in infertility treatment, the effects of HSG and ovarian stimulation cannot be ignored. Our review has demonstrated that many factors affecting TSH should be considered when evaluating a patient for mild SCH, including the timing and exact method of TSH measurement, as well as the age, diet, geographical location, and ethnicity of the patient. The review of the 28 studies considered here indicates that all reports of mild SCH contributing to miscarriage involved a diagnosis in early pregnancy, as opposed to before pregnancy. The question of whether TSH > 2.5 mIU/L is an appropriate cutoff value for the diagnosis of mild SCH remains controversial, and the essential problem is the handling of so‐called “gray area cases” of SCH (such as when TSH < 4.5 mIU/L). It is necessary to continue to study how mild SCH contributes to adverse events in infertility treatment and pregnancy.

## DISCLOSURES

*Conflict of interest*: Shuhei So is affiliated with the funded laboratory of Tawara IVF Clinic. Fumiko Tawara declares that they have no conflict of interest. *Human/Animal rights*: This article does not contain any studies with human and animal subjects performed by the any of the authors.
